# Construction of magnetic nanochains to achieve magnetic energy coupling in scaffold

**DOI:** 10.1186/s40824-022-00278-2

**Published:** 2022-08-06

**Authors:** Cijun Shuai, Xuan Chen, Chongxian He, Guowen Qian, Yang Shuai, Shuping Peng, Youwen Deng, Wenjing Yang

**Affiliations:** 1 Institute of Additive Manufacturing, Jiangxi University of Science and Technology, Nanchang, 330013 China; 2 State Key Laboratory of High Performance Complex Manufacturing, Central South University, Changsha, 410083 China; 3 College of Life Science and Technology, Huazhong University of Science and Technology, Wuhan, 430074 China; 4 The Key Laboratory of Carcinogenesis and Cancer Invasion of the Chinese Ministry of Education, Xiangya Hospital, Central South University, Changsha, 410078 China; 5 NHC Key Laboratory of Carcinogenesis of Hunan Cancer Hospital and the Affiliated Cancer Hospital of XiangyaSchool of MedicineSchool of Basic Medical Science, Cancer Research Institute, Central South University, Changsha, 410013 China; 6 School of Energy and Machinery Engineering, Jiangxi University of Science and Technology, Nanchang, 330013 China; 7 Department of Spine Surgery, Third Xiangya Hospital, Central South University, Changsha, 410013 China

**Keywords:** Magnetic microenvironment, Fe_3_O_4_ nanoparticles, Magnetic nanochains, Magnetic energy coupling, Bone scaffold

## Abstract

**Background:**

Fe_3_O_4_ nanoparticles are highly desired for constructing endogenous magnetic microenvironment in scaffold to accelerate bone regeneration due to their superior magnetism. However, their random arrangement easily leads to mutual consumption of magnetic poles, thereby weakening the magnetic stimulation effect.

**Methods:**

In this study, magnetic nanochains are synthesized by magnetic-field-guided interface co-assembly of Fe_3_O_4_ nanoparticles. In detail, multiple Fe_3_O_4_ nanoparticles are aligned along the direction of magnetic force lines and are connected in series to form nanochain structures under an external magnetic field. Subsequently, the nanochain structures are covered and fixed by depositing a thin layer of silica (SiO_2_), and consequently forming linear magnetic nanochains (Fe_3_O_4_@SiO_2_). The Fe_3_O_4_@SiO_2_ nanochains are then incorporated into poly l-lactic acid (PLLA) scaffold prepared by selective laser sintering technology.

**Results:**

The results show that the Fe_3_O_4_@SiO_2_ nanochains with unique core–shell structure are successfully constructed. Meanwhile, the orderly assembly of nanoparticles in the Fe_3_O_4_@SiO_2_ nanochains enable to form magnetic energy coupling and obtain a highly magnetic micro-field. The in vitro tests indicate that the PLLA/Fe_3_O_4_@SiO_2_ scaffolds exhibit superior capacity in enhancing cell activity, improving osteogenesis-related gene expressions, and inducing cell mineralization compared with PLLA and PLLA/Fe_3_O_4_ scaffolds.

**Conclusion:**

In short, the Fe_3_O_4_@SiO_2_ nanochains endow scaffolds with good magnetism and cytocompatibility, which have great potential in accelerating bone repair.

## Introduction

Recent scaffolds lack the capacity to effectively modulate cell growth or tissue reconstruction, resulting in slow bone regeneration and even failure of bone implantation [1–4]. It is well known that cells are magnetically sensitive due to the diamagnetism of cell membranes [5], and exposure to magnetic fields served to alter membrane flux and regulate ion channels and biochemical pathways [6, 7]. In this case, a series of cell behaviors will be mediated. Inspired by this, it is significant to construct an endogenous magnetic microenvironment in bone scaffold to mediate cell behaviors and tissue regeneration via magnetic stimulation.

As a typical magnetic material, Fe_3_O_4_ nanoparticles have been widely used in tissue engineering field due to their superior magnetic properties and good biocompatibility [8]. In addition, Fe_3_O_4_ nanoparticles enable to decompose into oxygen and iron in the body and can be easily removed from the body after degradation by oxygen transport and metabolism [9]. In our previous study [10], the introduction of Fe_3_O_4_ nanoparticles into bone scaffolds constructed an endogenous magnetic microenvironment that enhanced cell activity and accelerated new bone generation. It is worth noting that the random arrangement of Fe_3_O_4_ nanoparticles in scaffold easily leads to mutual repulsive of magnetic poles between adjacent nanoparticles, thereby weakening the magnetic strength. In this case, the scaffold cannot fully exert its regulation on cell behaviors.

The directional assembly of Fe_3_O_4_ nanoparticles into ordered nanochain structures is expected to solve the above problem. Under an external magnetic field, the internal magnetic dipole moment of Fe_3_O_4_ nanoparticles enable to be rapidly deflected to the direction of magnetic field [11, 12]. Moreover, the attractive magnetic dipole interaction will drive multiple Fe_3_O_4_ nanoparticles to assemble into ordered magnetic nanochain structures along with magnetic force lines [13, 14]. Compared with randomly arranged Fe_3_O_4_ nanoparticles, the magnetic nanochain structures can realize the magnetic energy coupling between the nanoparticles, thereby enhancing the magnetic strength [15–17]. Li et al. synthesized uniform linear cobalt nanochains with a coating layer of polyvinylpyrrolidone under an external magnetic field [18]. Wan et al. synthesized magnetic nanochains and fixated with a protective mesoporous silicon shell for osteoclast-targeted inhibition and heterogeneous nanocatalysis [19].

In this study, Fe_3_O_4_@SiO_2_ magnetic nanochains with core–shell structure were synthesized utilizing magnetic-field-guided interface co-assembly of nanoparticles. In short, Fe_3_O_4_ nanoparticles were firstly coated with a layer of protective SiO_2_ film. Then, the above products were aligned and assembled into nanochains under an external magnetic field. Thereafter, the nanochains were fixated by further deposition of SiO_2_ shell to permanently preserve their structure. Then, the Fe_3_O_4_@SiO_2_ nanochains were loaded into PLLA and prepared into porous PLLA/Fe_3_O_4_@SiO_2_ scaffold using selective laser sintering (SLS). The microscopic morphology, chemical composition, and physicochemical properties of Fe_3_O_4_@SiO_2_ were analyzed. The cell activity, osteogenic differentiation and mineralization abilities induced by the PLLA/Fe_3_O_4_@SiO_2_ scaffold were investigated and analyzed in detail. Moreover, the osteogenesis-related gene expressions of runt-related transcription factor-2 (Runx2), osteopontin (OPN), osteocalcin (OCN) and osterix (OSX) in cells co-cultured with the scaffold were assessed.

## Experimental sections

### Materials

Medical-grade PLLA powders were obtained from Shenzhen Polymtek Biomate-rial Co., Ltd. (Shenzhen, China). Fe_3_O_4_ nanoparticles with an average diameter of 100 nm, tetraothorsilicate (TEOS), sodium citrate dihydrate (C_6_H_5_Na_3_O_7_·2H_2_O), sodium hydroxide (NaOH) and concentrated ammonia solution (NH_3_·H_2_O, 28 wt.%) were purchased from Aladdin Chemistry Co. Ltd. (Shanghai, China). The above chemicals were applied as received without further purification.

### Synthesis of one-dimensional Fe3O4@SiO2 nanochains

**Fig. 1:**
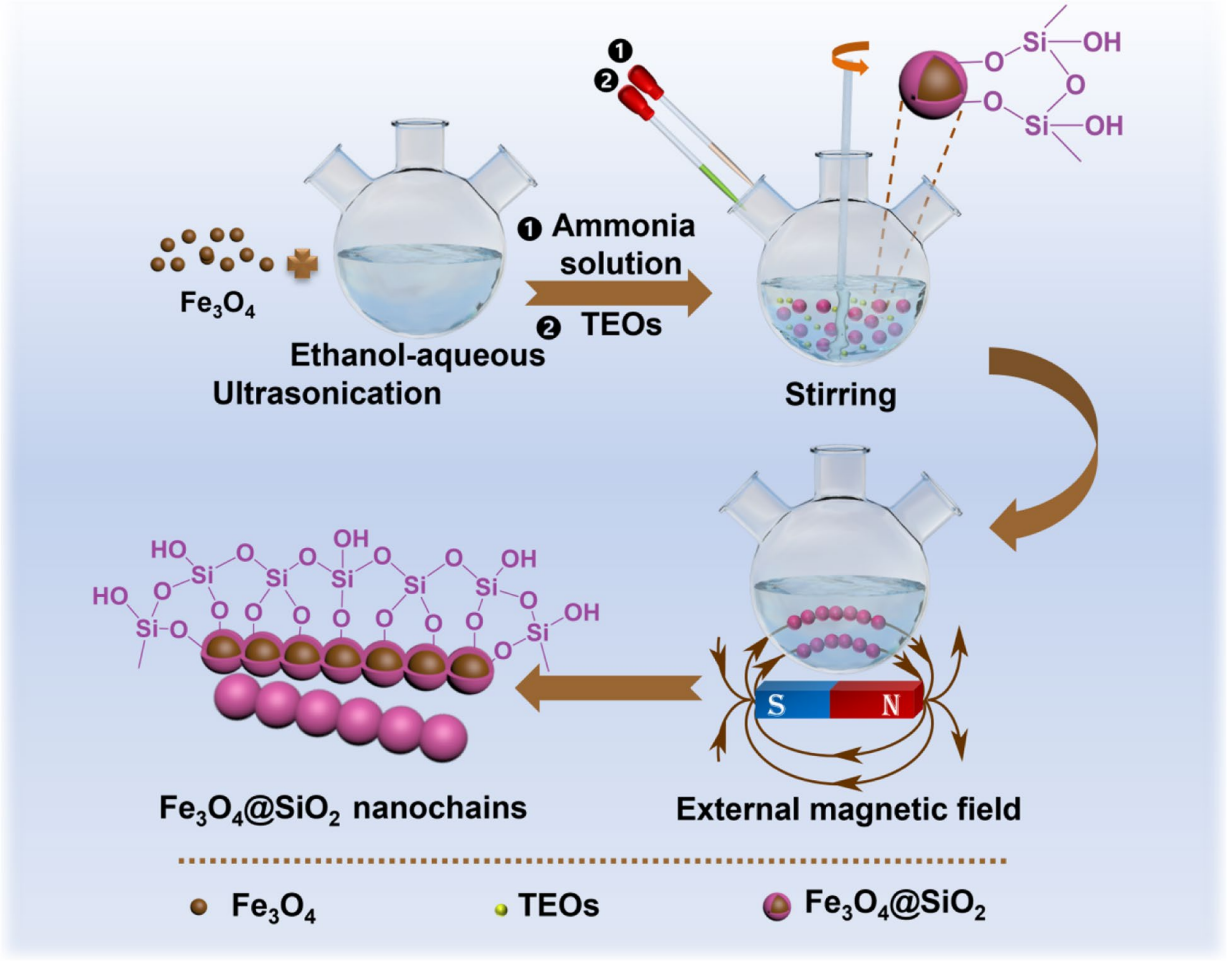
Synthesis of Fe_3_O_4_@SiO_2_ nanochains. N and S respectively represent the north pole and south pole of external magnetic field

The Fe_3_O_4_@SiO_2_ nanochains were prepared through the magnetic-field-guided interface co-assembly of Fe_3_O_4_ nanoparticles, as shown in Fig. 1. First, 20 mg of Fe3O4 nanoparticles was dispersed in 60 mL ethanol-aqueous solution by ultrasonication for 20 min. Subsequently, 5 mL of ammonia solution (28 wt.%) was dropped into the resulting solution with mechanical agitating (800 rpm) for 30 min. Then, the agitating speed was reduced to 300 rpm, and 10 mL of ethanol solution with TEOS (V_ethanol_:V_TEOS_ = 9:1) were dropped in the solution to coat Fe_3_O_4_ nanoparticles with a layer of protective SiO_2_ film in the early stage of the sol–gel reaction of TEOS. After reaction for 15 min, the above solution was exposed in a static magnetic field (55 mT-65 mT) for 2 min to induce the linear arrangement of Fe_3_O_4_ nanoparticles without stirring, forming numerous nanochain structures. After standing for another 10 min, the nanochain structures were further fixated with an addition SiO_2_ shell. Finally, the fixated core–shell Fe_3_O_4_@SiO_2_ magnetic nanochains were magnetically separated from the suspension, washed three times with ethanol and distilled water and dried in a vacuum oven.

### Preparation of scaffolds

For comparison, PLLA, PLLA/Fe_3_O_4_ and PLLA/Fe_3_O_4_@SiO_2_ scaffolds were respectively fabricated. Prior to fabricating, PLLA/Fe_3_O_4_@SiO_2_ and PLLA/Fe_3_O_4_ powders were prepared as follows. 4.6 g of PLLA powders and 0.4 g of Fe_3_O_4_@SiO_2_ nanochains or Fe_3_O_4_ nanoparticles were added to a beaker containing 30 mL of ethanol, in which the feeding mass ratio of Fe_3_O_4_@SiO_2_ or Fe_3_O_4_ to PLLA was effectively controlled at 8 wt.%. Then, the suspension was ultrasound for 30 min, following by vigorously stirring 1 h. Thereafter, the result suspensions were vacuum dried for 24 h at 65 °C, obtaining PLLA/Fe_3_O_4_@SiO_2_ or PLLA/Fe_3_O_4_ powders.

The representative PLLA/Fe_3_O_4_@SiO_2_ scaffold with honeycomb structure was fabricated via SLS technology. In detail, the PLLA/Fe_3_O_4_@SiO_2_ powders were paved on the powder bed and selectively laser scanned according to the designed three-dimensional model, with laser power at 2.5 W, scanning speed at 100 mm/s and scanning distance at 0.24 mm [20, 21]. The modeling platform gradually descended with each layer of powders was sintered until the scaffold was completely formed.

### Measurement and characterization

The morphologies of the Fe_3_O_4_@SiO_2_ nanochains were observed by transmission electron microscope (TEM, TALOS F200X, USA). The chemical structure, compositions and crystal structures of the Fe_3_O_4_@SiO_2_ or/and Fe_3_O_4_ powders were detected by X-ray photoelectron spectrometer (XPS, EscaLab 250Xi, USA), Fourier transform infra-red spectrometer (FTIR, FTIR 850, China) and X-ray diffractometer (XRD, D/MAX-RA, Japan). The magnetic properties of Fe_3_O_4_@SiO_2_ and Fe_3_O_4_ powders were evaluated using a vibrating sample magnetometer (VSM, LakeShore7404, USA). The mechanical properties were detected on a universal testing machine, with samples (6 × 5 × 3 mm^3^) for the compressive tests and dumbbell samples (L_0_ = 10.1 mm, h = 2.2 mm) for the tensile tests. The thermal behaviors of scaffolds were tested by a Pyris 1 thermal gravimetric analyzer (TGA, PerkinElmer, USA) under nitrogen at a heating rate of 20 °C/min. Water contact angle on PLLA, PLLA/Fe_3_O_4_ and PLLA/Fe_3_O_4_@SiO_2_ scaffolds were assessed using an optical contact angle meter (DM-501, Japan).

### Cytocompatibility

The PLLA, PLLA/Fe_3_O_4_, PLLA/Fe_3_O_4_@SiO_2_ scaffolds (φ 8 × 2 mm^3^) were sterilized by immersing in 70% ethanol solution for 2 h and irradiating with UV for 12 h. Then, the sterilized scaffolds were individually placed in 48-well culture plates. MG-63 cells (Sigma, Shanghai, China) were selected to investigate the cytocompatibility of scaffolds. MG-63 cells cultured in Dulbecco's Modified Eagle's Medium (DMEM) were washed with D-Hanks solution three times and were digested with trypsin. The resulting solution were centrifuged at 1000 r/min for 5 min. The cell suspension was diluted to 8 × 10^3^ cells/mL. All the cells were incubated in a humidified condition with 5% CO_2_ at 37 °C.

The cells at a density of 4 × 10^3^ cells/well were incubated on the surface of sterilized scaffolds in 48-well plates containing DMEM, in which the DMEM was updated daily. After incubating for 3 and 7 days, each cell-scaffold sample was washed with PBS three times, and then immersed in 5% glutaraldehyde for 30 min to fix cells. Thereafter, the cell-scaffold samples were dehydrated with 30%, 50%, 70%, 80%, 90%, 95% and absolute ethyl alcohol in sequence and dried for 12 h at room temperature. After coated with gold, the cell morphology on each scaffold was observed by scanning electron microscope (SEM).

Cell viabilities on the scaffolds were studied by a live/dead staining kit (Beyotime, China). At the specified time (3 and 7 days), the culture medium was removed, and the cells were detached from scaffolds and rinsed using PBS three times. Subsequently, the cells were stained with 2 μM Calcein-AM for 30 min at 37 °C. Finally, the stained cells were visualized using a fluorescence microscope (Olympus, Japan).

Cell proliferation was quantitatively assessed using Cell Counting Kit-8 (CCK-8, Beyotime, China) assay. The cell-scaffold samples were harvested from culture medium after 1, 4 and 7 days of cultivating. Then, the samples were washed with PBS and transferred into 96-well plates containing 100 μL CCK-8 reagent. After incubating for 2 h, the absorbance of the solution was detected utilizing a microplate reader (Thermal, USA) at 450 nm. Each group was carried out three parallel experiments.

The alkaline phosphatase (ALP) activity of the cells on scaffolds was determined to assess the osteogenic differentiation. After incubating for 7 days, the harvested cells from samples were washed with PBS 3 times and fixed with 4% paraformaldehyde for 15 min. Then, ALP staining kit (Beyotime, China) was dropped to stain the cells, and the stained cells were monitored using an inverted microscope (TE2000U, Japan).

The mineralization nodules of MG-63 cells cocultured with scaffolds were qualitatively investigated using Alizarin Red staining. The cells were seeded on scaffolds in 6-well plates for 7 days at a density of 1 × 10^4^ cells/mL. After that, the cell-scaffold samples were fixed using 4% paraformaldehyde and rinsed with PBS. Subsequently, the samples were stained with 0.04 M Alizarin Red for 10 min. After rinsing, the samples were observed under a light microscope.

Scaffold-mediated cell differentiation and osteogenesis were further studied. For this, the expression of several relative genes containing runt-related transcription factor-2 (Runx2), osteopontin (OPN), osteocalcin (OCN) and osterix (OSX) were detected with quantitative real-time polymerase chain reactions (RT-PCR). After incubating for 3 and 7 days, the RNA isolation of cells was employed using TRIzox reagent, and then the RNA was reverse transcribed to cDNA using PrimeScript 1st strand cDNA synthesis kit. Finally, the levels of Runx2, OPN, OCN and OSX were calculated using the 2^−ΔΔCt^ method. Each sample was analyzed three times.

### Statistical analysis

All data were conducted by Student’s *t*-test for independent samples and presented as means ± standard deviation, where **p < 0.01 and *p < 0.05 represented significant difference.

## Results

### Fe3O4@SiO2 nanochains

**Fig. 2:**
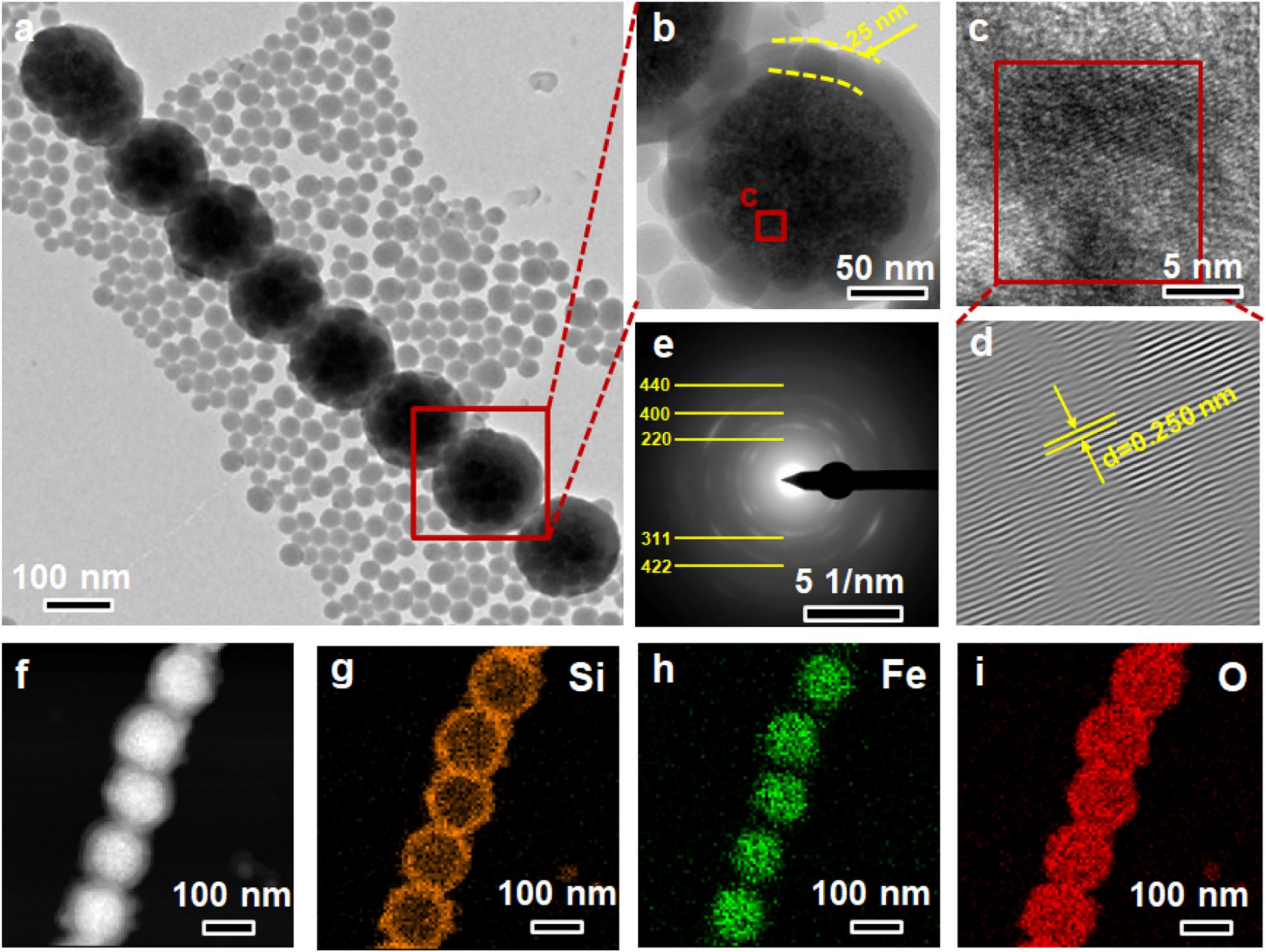
**a-c** TEM images, (**d**) the inverse-FFT image, (**e**) SAED patterns and (**f-i**) element mapping images of Fe_3_O_4_@SiO_2_ nanochains

The representative one-dimensional structure of the Fe_3_O_4_@SiO_2_ nanochains were observed using TEM. As shown in Fig. 2a and b, the Fe_3_O_4_@SiO_2_ nanochains presented a unique core–shell structure, in which Fe_3_O_4_ nanoparticles served as cores with a diameter of about 100 nm and SiO_2_ layers served as shells with a thickness of about 25 nm. Based on Fast Fourier transform (FFT, Fig. 2c and d), the interplanar distance between adjacent lattice fringes was 0.25 nm, which was in great agreement with the (311) lattice planes of Fe_3_O_4_. Moreover, the diffraction rings depicted in the selected area electron diffraction (SAED) patterns respectively corresponded to the (220), (311), (400), (440) and (422) lattice planes of cubic Fe_3_O_4_ (Fig. 2e). The Si, Fe and O elements were clearly exhibited in the elemental mapping images (Fig. 2f-i). Particularly, the distribution of Si and Fe elements further confirmed the shell-core structure of the nanochains. The results confirmed that the Fe_3_O_4_ nanoparticles could be induced to align in a nanochain though magnetic dipolar interaction under external magnetic field.

**Fig. 3:**
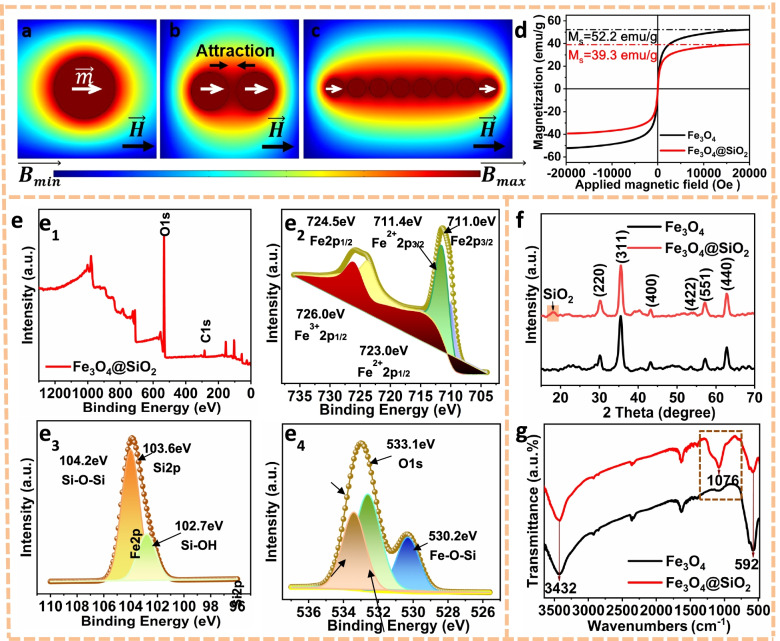
**a** Magnetic field distribution around a Fe_3_O_4_ nanoparticle. The attractive (**b**) dipole–dipole forces between two adjacent nanoparticles drive the formation of nanochains along the magnetic field (**c**). **d** Magnetization curves, (**e**) XPS spectra of Fe_3_O_4_@SiO_2_ nanochains. (e_1_) XPS survey spectrum along with the spectra of (e_2_) Fe2p, (e_3_) Si2p and (e_4_) O1s. (f) XRD patterns, (g) FTIR spectra

Magnetic field distribution around single Fe_3_O_4_ nanoparticle and nanochain in the same direction of external magnetic field was analyzed using finite element method (COMSOL Multiphysics), as shown in Fig. 3a-c. It could be seen that the magnetic dipole moment of a Fe_3_O_4_ nanoparticle reached a saturated value under the adequately strong magnetic field (Fig. 3a). When the centerlines of two adjacent nanoparticles were aligned with the direction of the external magnetic field, the dipole–dipole interaction was attractive (Fig. 3b). In the case of the interaction energy was large enough to overcome thermal fluctuations, the magnetic dipole–dipole force drove the self-assembly of nanoparticles into nanochain along the dipole moment (Fig. 3c). In this condition, the dipole–dipole coupling reached the maximum, and the magnetism of nanochain could be regarded as the magnetic energy coupling among multiple nanoparticles. The magnetic strength of nanochain was higher than that of randomly arranged Fe_3_O_4_ nanoparticles.

The magnetic properties of Fe_3_O_4_ nanoparticles and Fe_3_O_4_@SiO_2_ nanochains were detected and presented in Fig. 3d. It could be seen that both nanoparticles and nanochains exhibited superior magnetism, which was an important advantage for their applicability in biomedicine [22, 23]. The saturation magnetization of Fe_3_O_4_ was 52.2 emu/g. The relatively low saturation magnetization of Fe_3_O_4_@SiO_2_ was due to the introduction of non-magnetic SiO_2_ shells decreased the weight ratio of Fe_3_O_4_ in Fe_3_O_4_@SiO_2_. This phenomenon was also discovered by M. Tarhini and A. Bitar et al. [24, 25].

XPS spectra of Fe_3_O_4_@SiO_2_ nanochains were presented in Fig. 3e. The typical Fe2p, O1s and Si2p peaks were clearly observed (Fig. 3 e1). In detail, the peaks centered at 711.4, 723.0 and 726.0 eV were respectively corresponded to Fe^2+^2p_3/2_, Fe^2+^2p_1/2_ and Fe^3+^2p_1/2_ of Fe_3_O_4_ (Fig. 3 e2), while the peaks centered at 102.7 and 104.2 eV were assigned to Si–OH and Si–O-Si of SiO_2_ (Fig. 3 e3) [26]. It was worth noting that the binding energy of O1s was 533.1 eV (Fig. 3 e4), which was higher than that of Fe_3_O_4_ (529.6 eV) by 3.5 eV. This was mainly due to the formation of Fe–O-Si chemical bond (530.2 eV) decreased the electronic density of O binding Fe, resulting in the chemical shift of binding energy of O1s. Moreover, the coexistence of Si–O-Si and Fe–O-Si verified the coating of SiO_2_ on the nanochains.

The XRD patterns of Fe_3_O_4_ nanoparticles and Fe_3_O_4_@SiO_2_ nanochains were exhibited in Fig. 3f. There were typical diffraction peaks of (200), (311), (400), (422), (511) and (440) planes, which corresponded to Fe_3_O_4_ presented in both patterns, confirming that the crystal structure of Fe_3_O_4_ nanoparticles were completely preserved during the synthesis of Fe_3_O_4_@SiO_2_. Compared with Fe_3_O_4_, a new broad diffraction at around 20° appeared in Fe_3_O_4_@SiO_2_, which was attributed to the amorphous SiO_2_ shell [27]. In the FTIR spectrum (Fig. 3g), the absorption peak at 1076 cm^−1^ in Fe_3_O_4_@SiO_2_ was adscripted to Si–O bond while the peak at 592 cm^−1^ was attributed to Fe–O bond [28–30], which further confirmed that the core–shell structure of Fe_3_O_4_@SiO_2_ nanochains.

### Physical and chemical properties

**Fig. 4:**
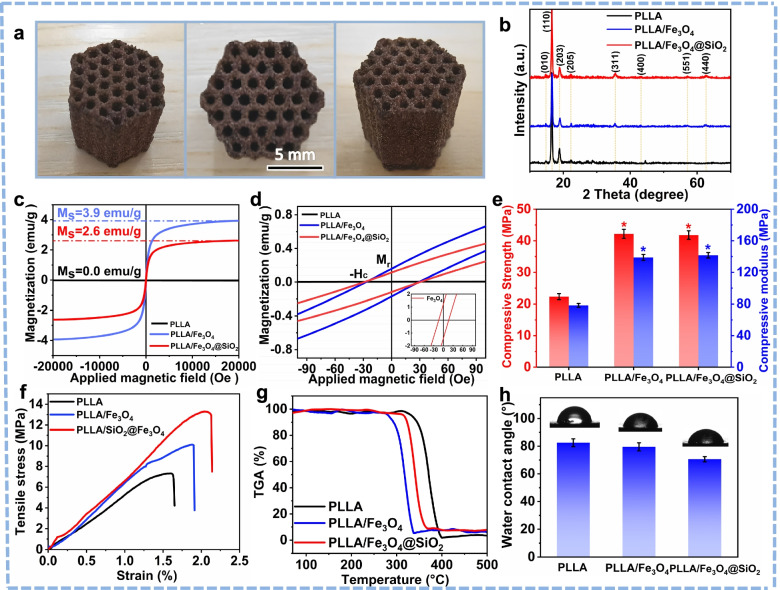
**a** Optical graphs of SLS prepared honeycomb scaffold. **b** XRD patterns. **c** Magnetization curves. **d** Magnetization behaviors at magnetic field from -90 to 90 Oe. **e** Compressive strength and compressive modulus (n = 5, *p < 0.05), (**f**) tensile stress–strain curves, (**g**) TGA curves and (**h**) hydrophilicity of PLLA, PLLA/Fe_3_O_4_ and PLLA/Fe_3_O_4_@SiO_2_ scaffolds

The porous scaffold with honeycomb structure was shown in Fig. 4a. The pore size of the scaffold was 800 ± 50 μm, which was proven to be beneficial to cell adhesion and climbing growth [31, 32]. The phase composition of the scaffolds was assessed using XRD (Fig. 4b). It can be clearly observed that the diffraction peaks belonged to (010), (110), (203) and (205) planes of PLLA [33]. By contrast, the new diffraction peaks corresponding to (311), (400), (551) and (440) crystal planes confirmed the spinel structure of Fe_3_O_4_ in PLLA/Fe_3_O_4_ and PLLA/Fe_3_O_4_@SiO_2_ scaffolds [34].

The magnetic behaviors of the PLLA, PLLA/Fe_3_O_4_ and PLLA/Fe_3_O_4_@SiO_2_ scaffolds were shown in Fig. 4c and d. It was clearly seen that the introduction of Fe_3_O_4_ and Fe_3_O_4_@SiO_2_ endowed the non-magnetic PLLA scaffold favorable magnetic properties. This was conducive to construct magnetic microenvironment in scaffold, which was expected to enhance cell viability and promote cell growth through magnetic stimulation.

Mechanical properties of the scaffolds were evaluated via compressive and tensile tests, with the results shown in Fig. 4e and f. The compressive strength and modulus of PLLA scaffolds were only 22.3 ± 0.9 MPa and 78.4 ± 6.2 MPa. Encouragingly, the compressive strength and modulus of the PLLA/Fe_3_O_4_@SiO_2_ scaffolds were 41.8 ± 1.6 MPa and 142.6 ± 8.5 MPa, which were increased by 87.4% and 80.6% compared with PLLA scaffolds. Moreover, the PLLA/Fe_3_O_4_@SiO_2_ scaffolds also exhibited much higher the tensile strength and strain than those of PLLA and PLLA/Fe_3_O_4_ scaffolds, which were 13.34 ± 1.2 MPa and 2.13%, respectively. The high mechanical properties of PLLA/Fe_3_O_4_@SiO_2_ scaffolds were attributed to that the Fe_3_O_4_@SiO_2_ nanochains acted as rigid reinforcement to enhance the stress transfer efficiency in the matrix.

The TGA measurement were performed to analyze the thermal decomposition and the corresponding residual weight of scaffolds (Fig. 4g). The slight weight loss below 300 °C of the scaffolds was related to the evaporation of adsorbed water molecules. Obviously, the thermal decomposition temperature of PLLA scaffolds was about 300 ~ 400 °C, while the range of decomposition temperature leftward shifted and narrowed after adding Fe_3_O_4_ and Fe_3_O_4_@SiO_2_. This confirmed that the addition of Fe_3_O_4_ and Fe_3_O_4_@SiO_2_ catalyzed the thermal decomposition of PLLA. Additionally, the residual weight of the PLLA/Fe_3_O_4_ and PLLA/Fe_3_O_4_@SiO_2_ scaffolds was 7.4 wt.% and 7.6 wt.%, respectively, which was close to the nominal content (8 wt.%) of Fe_3_O_4_ and Fe_3_O_4_@SiO_2_ introduced into PLLA matrix.

Generally, a scaffold with favorable hydrophilicity is more conducive to cell adhesion [35]. The hydrophilicity of PLLA, PLLA/Fe_3_O_4_ and PLLA/Fe_3_O_4_@SiO_2_ scaffolds was investigated via water contact angle test. As shown in Fig. 4h, the contact angle on the PLLA scaffold was 86.2 ± 2.8°. By contrast, the contact angle decreased after the adding Fe_3_O_4_ nanoparticles, indicating the improvement of hydrophilicity. This could be attributed to the presence of hydroxyl groups on the Fe_3_O_4_ nanoparticles in aqueous environment. Moreover, the PLLA/Fe_3_O_4_@SiO_2_ scaffolds exhibited the best hydrophilicity, which was mainly due to the silanol groups of SiO_2_ absorbed water molecule via hydrogen bonding.

### Cytocompatibility

**Fig. 5:**
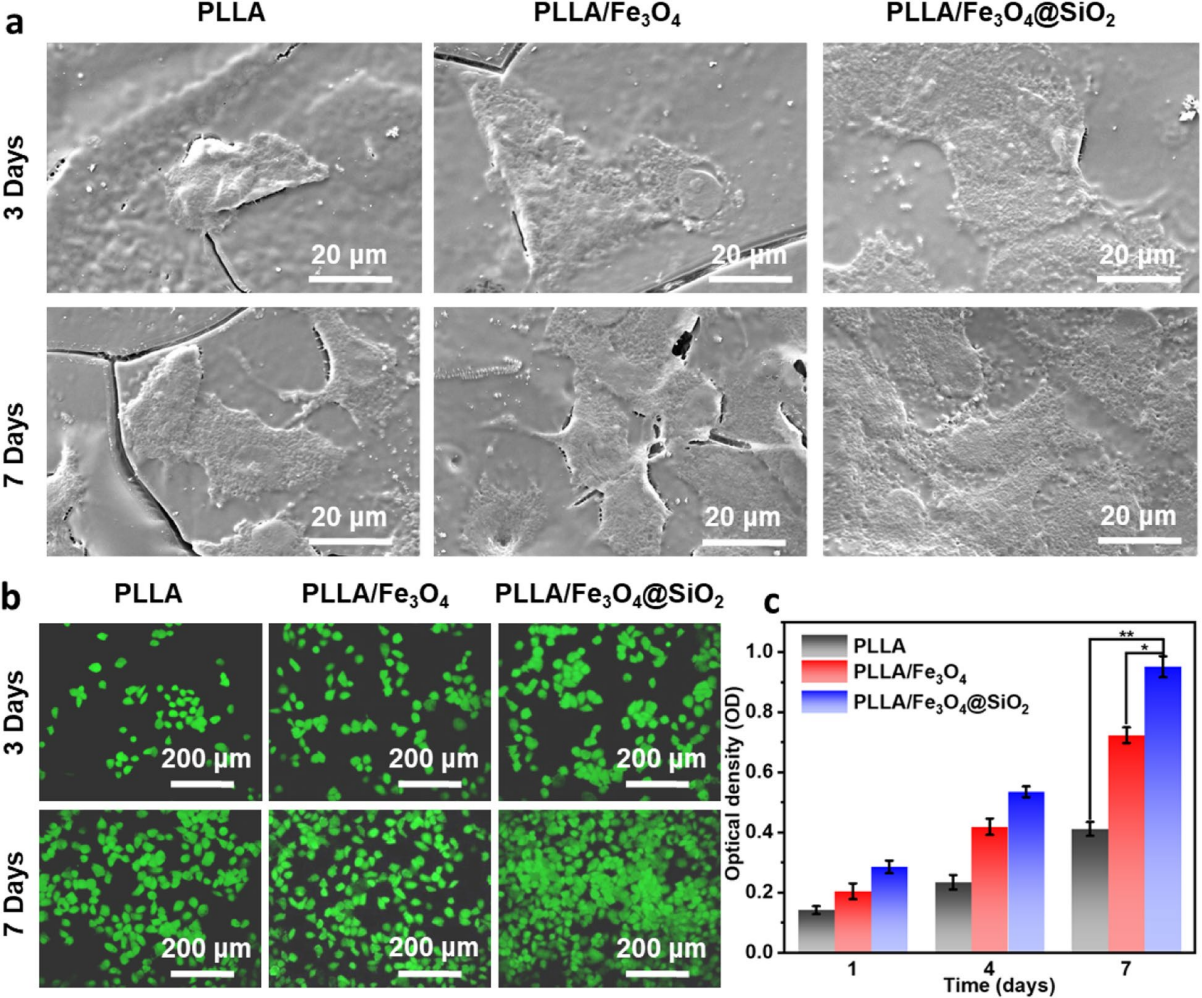
**a** Adhesion morphologies of MG-63 cells on PLLA, PLLA/Fe_3_O_4_ and PLLA/Fe_3_O_4_@SiO_2_ scaffolds. **b** Fluorescence images of cells cultured after 3 and 7 days on the scaffolds. **c** Cell proliferation on the scaffolds (n = 5, *p < 0.05, **p < 0.01)

The cytocompatibility of scaffolds is a necessary and crucial element in the bone repair process because it determines whether cells can adhere, grow, and proliferate on the scaffold [36, 37]. Herein, the cytocompatibility of the PLLA, PLLA/Fe_3_O_4_ and PLLA/Fe_3_O_4_@SiO_2_ scaffolds were assessed. As shown in Fig. 5a, cells adhered well on all scaffolds, indicating that the PLLA, PLLA/Fe_3_O_4_ and PLLA/Fe_3_O_4_@SiO_2_ scaffolds had good biocompatibility. Particularly, better cell adhesion morphology displayed on PLLA/Fe_3_O_4_@SiO_2_ scaffold than that on PLLA and PLLA/Fe_3_O_4_ scaffolds at the same time point. Moreover, the cells completely expanded and essentially presented normal topological configuration on PLLA/Fe_3_O_4_@SiO_2_ scaffold after 7 days of cultivation, indicating that the Fe_3_O_4_@SiO_2_ nanochains in scaffold were more conducive to cell adhesion and expansion.

Cell viability is also an important indicator for evaluating the cytocompatibility of the scaffold [38]. To investigate the cells viability induced by the PLLA, PLLA/Fe_3_O_4_ and PLLA/Fe_3_O_4_@SiO_2_ scaffolds, the cells were strained with calcein AM. Normally, calcein AM only stains living cells, because calcein AM as a dye can be transformed into a membrane impermeable fluorescent analogue by the cell esterases, and the fluorescence will leak out when the cell membrane is completely damaged [39, 40]. As shown in Fig. 5b, the density of living cells in PLLA, PLLA/Fe_3_O_4_ and PLLA/Fe_3_O_4_@SiO_2_ scaffold groups was significantly enhanced with time, confirming that all scaffolds possessed the ability to enhance cell activity. Notably, the cells increased exponentially from 3 to 7 days with the highest density observing in the cells which cocultured with PLLA/Fe_3_O_4_@SiO_2_ scaffold, indicating that the Fe_3_O_4_@SiO_2_ nanochains in scaffold significantly enhanced cell viability and promoted cell proliferation.

Cell proliferation is one of the important physiological functions of living cells. To quantitatively study the cell proliferation capacity on the PLLA, PLLA/Fe_3_O_4_ and PLLA/Fe_3_O_4_@SiO_2_ scaffolds, the CCK-8 assay was carried out (Fig. 5c). It could be clearly seen that the optical density (OD) value of cells on all the scaffolds increased significantly with incubating time. Compared with the PLLA scaffold, higher OD value of cells presented on the PLLA/Fe_3_O_4_ scaffold, indicating that the Fe_3_O_4_ nanoparticles in the scaffold promoted cell proliferation. Especially, the OD value of cells on PLLA/Fe_3_O_4_@SiO_2_ scaffold was markedly higher than that on PLLA/Fe_3_O_4_ scaffold, indicating that the Fe_3_O_4_@SiO_2_ nanochains further promoted cell proliferation.

**Fig. 6:**
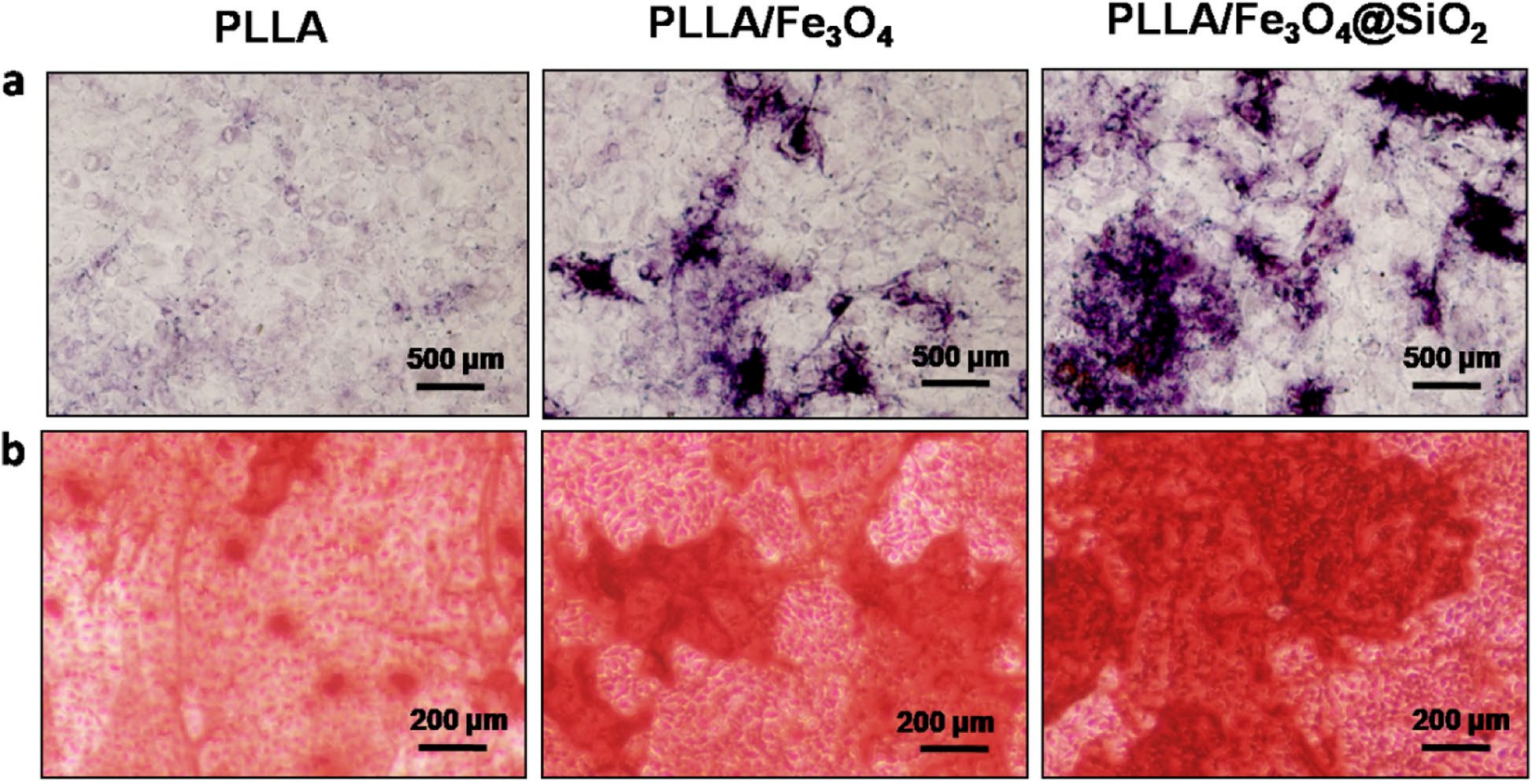
**a** The ALP and (**b**) Alizarin Red staining of cells on PLLA, PLLA/Fe_3_O_4_ and PLLA/Fe_3_O_4_@SiO_2_ scaffolds after 7 days of cultivation

As one of the early indicators of osteogenic differentiation [41], ALP activity of cells cultured on the scaffolds for 7 days was qualitatively analyzed (Fig. 6a). It could be clearly seen that the cells cocultured with the PLLA/Fe_3_O_4_@SiO_2_ scaffold samples exhibited higher ALP activity than PLLA and PLLA/Fe_3_O_4_ samples. As one of the late markers of osteogenic differentiation [42], the Alizarin Red staining was performed to assess the extracellular matrix mineralization of cells cocultured with the scaffolds for 7 days (Fig. 6b). As expected, there were obvious red precipitates in all scaffold groups. It was worth noting that the mineral deposition was significantly enhanced in PLLA/Fe_3_O_4_@SiO_2_ samples compared to other groups, mainly due to the stronger magnetic stimulation effect of Fe_3_O_4_@SiO_2_ nanochains. The above results demonstrated that the Fe_3_O_4_@SiO_2_ nanochains in scaffold markedly enhanced cell activity and promoted cell proliferation, differentiation, and mineralization.

**Fig. 7:**
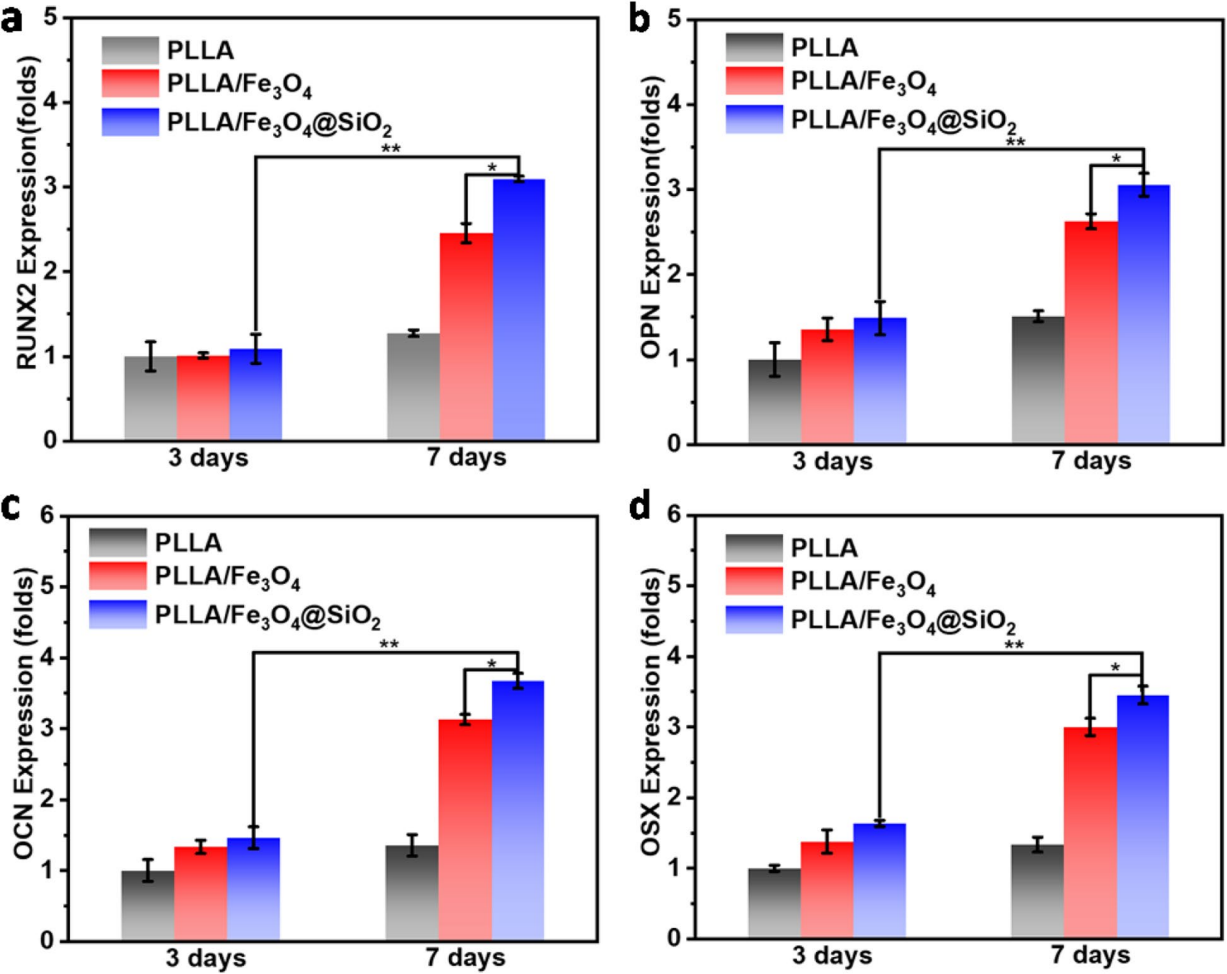
The expressions of (**a**) RUNX2, (**b**) OPN, (**c**) OCN and (**d**) OSX on the scaffolds after 3 and 7 days of culture (n = 5, *p < 0.05, **p < 0.01)

The bone-related gene expressions including RUNX2, OPN, OCN and OSX on PLLA, PLLA/Fe_3_O_4_ and PLLA/Fe_3_O_4_@SiO_2_ scaffolds were investigated (Fig. 7). From an overall perspective, the expression levels of RUNX2, OPN, OCN and OSX on day 7 were greatly higher than on day 3. Especially, the expression level of them on the PLLA/Fe_3_O_4_@SiO_2_ scaffold were markedly higher than that on PLLA/Fe_3_O_4_ and PLLA scaffolds at any time. The results showed that the Fe_3_O_4_@SiO_2_ nanochains provided a more favorable magnetic microenvironment for cell differentiation than Fe_3_O_4_ nanoparticles in scaffolds, confirming that the superior capability of Fe_3_O_4_@SiO_2_ nanochains to promote cell differentiation.

## Discussion

It is well known that various cells, such as mesenchymal stem cells, osteoblasts, and endothelial cells are magnetically sensitive due to the diamagnetism of cell membranes [43]. Inspired by these, researchers have applied different external magnetic fields to study the roles of magnetic stimulation in bone repair in recent years [44, 45]. It was found that the external magnetic fields could induce a series of cell behaviors by regulating cell surface receptors and signaling pathways via magnetic stimulations, thereby accelerating new bone regeneration or inhibiting osteoclast resorption. However, the need of magnetic field generators limits the clinical application of magnetic stimulation to a certain extent.

To solve the above problem, it would be an effective means to construct an endogenous magnetic microenvironment in bone scaffolds by introducing magnetic materials. As a highly biocompatible and magnetic materials, Fe_3_O_4_ nanoparticles have received clinical approval from the Food and Drug Administration. The scaffolds loaded with Fe_3_O_4_ nanoparticles indeed effectively enhanced cell viability and promoted cell proliferation [46, 47]. However, the random arrangement of Fe_3_O_4_ nanoparticles in the scaffolds greatly compromised their positive magnetic stimulation effects, due to the mutual repulsive between adjacent magnetic dipoles.

In present study, we constructed Fe_3_O_4_@SiO_2_ nanochains with uniform shell-core structure by magnetic-field-guided interface co-assembly of Fe_3_O_4_ nanoparticles (Fig. 2). The simulation analysis results of magnetic field distribution proved the orderly assembly of Fe_3_O_4_ nanoparticles in the Fe_3_O_4_@SiO_2_ nanochains formed magnetic energy coupling and obtained a highly magnetic micro-field (Fig. 3a-c). The results are consistent with the analysis of Yin Yadong's team [48–50]. From the results of magnetic tests, the Fe_3_O_4_@SiO_2_ nanochains still preserved the superior superparamagnetism of Fe_3_O_4_ nanoparticles. The good magnetism and high surface areas endowed Fe_3_O_4_@SiO_2_ nanochains with great potential for use in biomedicine.

**Fig. 8:**
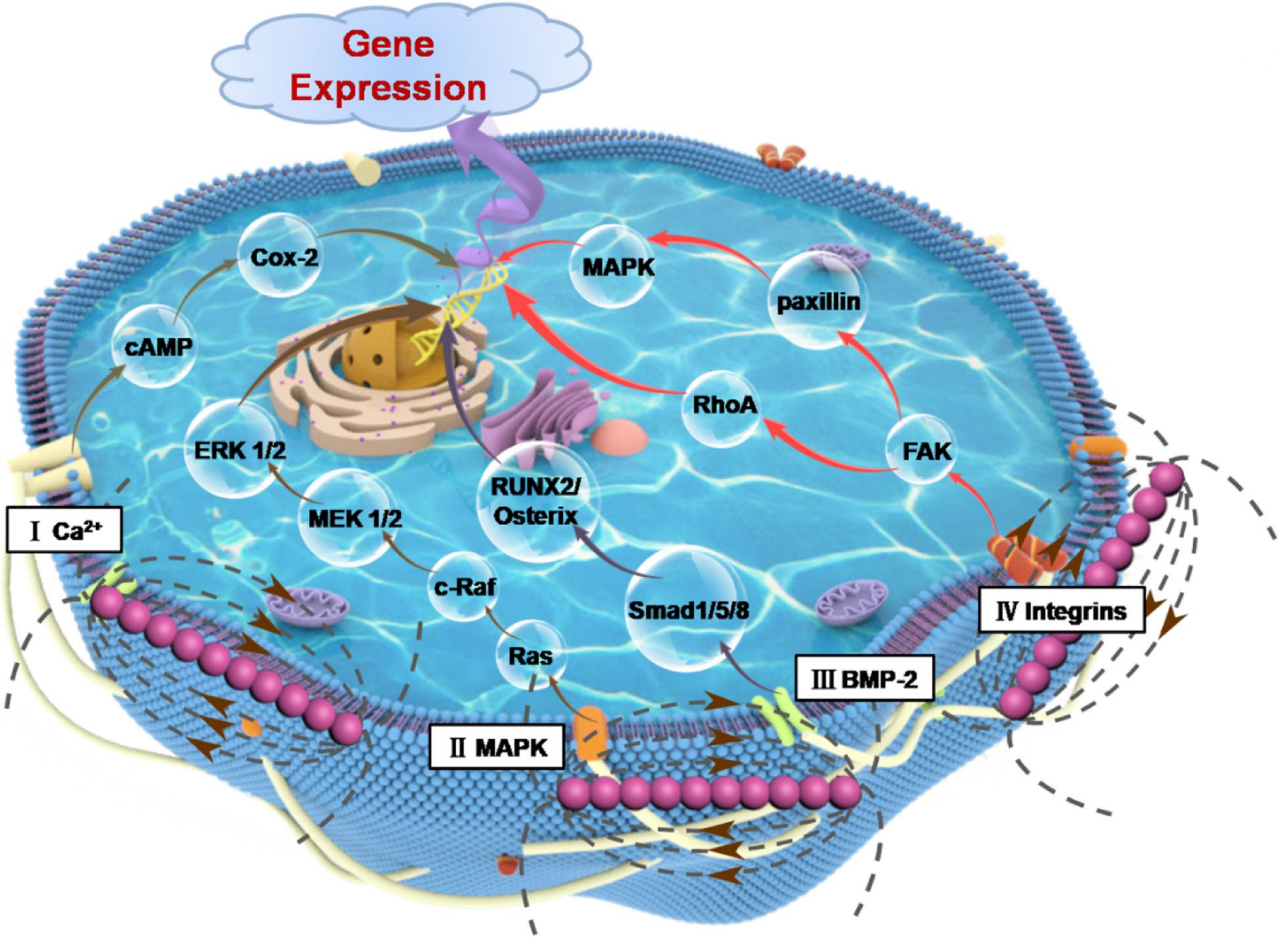
Schematic illustration of Fe_3_O_4_@SiO_2_ magnetic nanochains in up-regulating osteogenesis-related gene expressions

To better understand the biological advantages of Fe_3_O_4_@SiO_2_ nanochains in scaffolds, a series of in vitro cell experiments were performed. Compared to PLLA/Fe_3_O_4_ scaffolds, PLLA/Fe_3_O_4_@SiO_2_ scaffolds are more conducive to cell adhesion and expansion, especially further enhancing cell viability, proliferation, differentiation, mineralization, and bone-related gene expressions (Figs. 5, 6 and 7). It could be attributed to the stronger magnetic stimulation effect of Fe_3_O_4_@SiO_2_ nanochains. In terms of mechanism, the orderly assembly of Fe_3_O_4_ nanoparticles obtained magnetic energy coupling, resulting in a highly micro-field that stimulated the surrounding cells to respond (Fig. 8). In this case, the membrane flux of the diamagnetic cell membrane would be modified. Moreover, the strong magnetic singles would activate receptors on the cell membrane, thereby modulating a series of signaling pathways including Ca^2+^ channels, mitogen-activated protein kinase (MAPK), bone morphogenetic protein-2 (BMP-2) and integrins [6, 10, 41, 51]. Then, the corresponding downstream transcription factors were regulated, and consequently osteogenesis-related gene expressions of RUNX2, OPN, OCN and OSX were up-regulated. Hence, the Fe_3_O_4_@SiO_2_ nanochains in scaffold possessed great potential in accelerating bone repair.

## Conclusions

A magnetic-field-guided interface co-assembly of Fe_3_O_4_ nanoparticles had been demonstrated to rationally synthesis unique Fe_3_O_4_@SiO_2_ nanochains. The obtained Fe_3_O_4_@SiO_2_ nanochains exhibited high magnetic susceptibility and excellent magnetic induction intensity. Importantly, the superior magnetic properties of nanochains enhanced the interaction between PLLA/Fe_3_O_4_@SiO_2_ scaffold and cells. As a result, the nanochains in scaffold effectively enhanced cell activity, proliferation, differentiation, and mineralization as well as bone-related gene expressions. These findings confirmed the superparamagnetic scaffold incorporated with Fe_3_O_4_@SiO_2_ nanochains could accelerate the repair of bone defect.

## Data Availability

The datasets used and/or analysed during the current study are available from the corresponding author on reasonable request.

## Abbreviations

PLLA: Poly l-lactic acid
SiO_2_: Silica
SLS: Selective laser sintering
TEOS: Tetraothorsilicate
TEM: Transmission electron microscope
XPS: X-ray photoelectron spectrometer
FTIR: Fourier transform infra-red spectrometer
XRD: X-ray diffractometer
VSM: Vibrating sample magnetometer
DMEM: Dulbecco's Modified Eagle's Medium
SEM: Scanning electron microscope
CCK-8: Cell Counting Kit-8
ALP: Alkaline phosphatase
Runx2: Runt-related transcription factor-2
OPN: Osteopontin
OCN: Osteocalcin
OSX: Osterix
